# The Plant Proteinase Inhibitor* CrataBL* Plays a Role in Controlling Asthma Response in Mice

**DOI:** 10.1155/2018/9274817

**Published:** 2018-10-01

**Authors:** Anelize Sartori Santos Bortolozzo, Adriana Palmeira Dias Rodrigues, Fernanda Magalhães Arantes-Costa, Beatriz Mangueira Saraiva-Romanholo, Flávia Castro Ribas de Souza, Thayse Regina Brüggemann, Marlon Vilela de Brito, Rodrigo da Silva Ferreira, Maria Tereza dos Santos Correia, Patrícia Maria Guedes Paiva, Carla Máximo Prado, Edna Aparecida Leick, Maria Luiza Vilela Oliva, Milton de Arruda Martins, Viviane Christina Ruiz-Schutz, Renato Fraga Righetti, Iolanda de Fátima Lopes Calvo Tibério

**Affiliations:** ^1^Departamento de Clínica Médica, Faculdade de Medicina da Universidade de São Paulo, 01246-903 São Paulo, SP, Brazil; ^2^Universidade Cidade de São Paulo, São Paulo, SP, Brazil; ^3^Universidade do Estado de Minas Gerais, 04044-020 Passos, MG, Brazil; ^4^Departamento de Bioquímica, Universidade Federal de São Paulo, 04044-020 São Paulo, SP, Brazil; ^5^Departamento de Bioquímica, Universidade Federal de Pernambuco, 50670-910 Recife, PE, Brazil; ^6^Departamento de Ciências Biológicas, Universidade Federal de São Paulo, 11015-020 Santos, SP, Brazil

## Abstract

*Background. CrataBL* is a protein isolated from* Crataeva tapia* bark. It has been shown to exhibit several biological properties, including anti-inflammatory, analgesic, antitumor, and insecticidal activities. There are no studies evaluating the role of* CrataBL* in experimental asthma models.* Aim*. To evaluate the effects of* CrataBL* on lung mechanics, inflammation, remodeling, and oxidative stress activation of mice with allergic pulmonary inflammation.* Materials and Methods*. BALB/c mice (6-7 weeks old, 25-30g) were divided into four groups: nonsensitized and nontreated mice (C group, n=8); ovalbumin- (OVA-) sensitized and nontreated mice (OVA group, n=8); nonsensitized and* CrataBL*-treated mice (C+CR group, n=8); OVA-sensitized and* CrataBL*-treated mice (OVA+CR group, n=8). We evaluated hyperresponsiveness to methacholine, bronchoalveolar lavage fluid (BALF), pulmonary inflammation, extracellular matrix remodeling, and oxidative stress markers.* Results. CrataBL *treatment in OVA-sensitized mice (OVA+CR group) attenuated the following variables compared to OVA-sensitized mice without treatment (OVA group) (all p<0.05): (1) respiratory system resistance (Rrs) and elastance (Ers) after methacholine challenge; (2) total cells, macrophages, polymorphonuclear cells, and lymphocytes in BALF; (3) eosinophils and volume fraction of collagen and elastic fibers in the airway and alveolar wall according to histopathological and morphometry analysis; (4) IL-4-, IL-5-, IL-13-, IL-17-, IFN-*γ*-, MMP-9-, TIMP-1-, TGF-*β*-, iNOS-, and NF-kB-positive cells and volume of 8-iso-PGF2*α* in airway and alveolar septa according to immunohistochemistry; and (5) IL-4, IL-5, and IFN-*γ* according to an ELISA.* Conclusion. CrataBL* contributes to the control of hyperresponsiveness, pulmonary inflammation, extracellular matrix remodeling, and oxidative stress responses in an animal model of chronic allergic pulmonary inflammation.

## 1. Introduction

Asthma is a chronic inflammatory airway disease characterized by allergen-induced airway hyperresponsiveness and pulmonary inflammation, as well as airflow limitation and remodeling [1]. Its prevalence has increased considerably over the past three decades, representing a major global health problem [2].

The symptoms of asthma, such as wheezing, breathlessness, chest tightness, coughing, and expiratory flow limitation, vary in intensity over time [1]. Inhaled or oral corticosteroids are widely used, acting as powerful anti-inflammatories [1, 3, 4]. However, some patients remain symptomatic, despite optimal corticosteroid therapy, and there is evidence of persistent airway and distal lung inflammation: corticosteroids seem to have little, if any, effect on airway remodeling [4, 5]. In addition, there are side-effects associated with long term use of corticosteroids, including hypertension, osteoporosis, cataracts, gastrointestinal disorders, and type 2 diabetes [6]. Therefore, new therapies are currently being studied to improve regulation of the different processes involved in the pathophysiology of asthma [4, 7]. Biologic medications are increasingly given to treatment-resistant patients, but they can be costly, have complex dosing and management, and are not widely available around the world [8–10]. There is evidence that proteinase inhibitors play an important role in inhibiting proteinase activities during homeostasis, inflammation, tissue injury, and cancer progression. Their emerging role as a significant contributor to inflammatory pathologies has led to interest in their potential as drugs for disease treatment [11, 12]. In models of asthma, some proteinase inhibitors were tested, and their potential anti-inflammatory effects in the airways have been demonstrated [13].

*Crataeva tapia (Capparidaceae) *is a tree broadly distributed across Brazil, occurring in the Pluvial Tropical Atlantic Forest and* Pantanal* region. The* Crataeva tapia Bark Lectin* (*CrataBL, *resembling the Kunitz-type plant proteinase inhibitors family) is a bifunctional glycoprotein isolated from the bark of* Crataeva tapia* that exhibits lectin activity and inhibits trypsin serine proteinase (Kiapp = 43 *μ*M); the coagulation factor Xa (Kiapp = 8.2 *μ*M).* CrataBL's* biological effects include impairment of the intrinsic pathway of the coagulation cascade [14], anti-inflammatory and analgesic activities [15], insecticidal effects [16], and antitumor activities [12].

Currently, there are no studies evaluating the role of* CrataBL* in experimental asthma. Therefore, our aim was to study the effects of* CrataBL* on lung mechanics, inflammation, remodeling, and oxidative stress activation in airways and alveolar walls in mice with allergic pulmonary inflammation.

## 2. Materials and Methods

### 2.1. Purification of* CrataBL*

*CrataBL* was purified following previously described methodology [12]. Powdered bark (10 x* g*) was suspended in 0.15 M NaCl (100 mL). A clear supernatant (crude extract) was obtained after homogenization in a magnetic stirrer (16 h at 4°C), followed by filtration through gauze and centrifugation (4000 ×* g*, 15 min). The extract was evaluated for protein concentration and hemagglutinating activity. Soluble proteins in the crude extract were fractionated with ammonium sulfate according to Green and Hughes [17], and 30–60% of the precipitate fraction (30–60 F) was submitted to dialysis (3.5 KDa cut-off membrane, 4°C) against distilled water (2 hours) followed by 10 mM citrate-phosphate buffer pH 5.5 (2 hours). The 30–60 F was loaded (11 mg of protein, hemagglutinating activity of 1024) onto a CM-cellulose (*Sigma–Aldrich, USA*) column (5.2 cm × 1.6 cm), equilibrated with 10 mM citrate-phosphate buffer pH 5.5 at a flow rate of 20 mLh−1. Unabsorbed proteins were eluted with equilibrating solution until the absorbance at 280 nm was lower than 0.05. Next, the adsorbed hemagglutinating activity (*CrataBL*) was eluted with 0.5 M NaCl [18].

Protein homogeneity was confirmed by hydrophobicity chromatography on a *μ*-Bondapack C18 column (Beckman Ultrasphere-15 × 0.5 cm) and mass spectrometry. The column was equilibrated with trifluoroacetic acid (TFA) 0.1% (v/v) in Milli-Q water (Solvent A). Protein was eluted with 90% acetonitrile gradient (v/v) in 0.1% TFA (v/v) in Milli-Q water (Solvent B) (t =0.1 min, B=5%, t=5 min, B=5%, t=30 min, % B=40, t=50 min, % B=50, t=60 min B=100%; t=65-68 min, and B=0%) for 60 min at a flow rate of 0.7 mL/min. The elution profile was monitored at 280 nm.

The protein preparation was analyzed by MALDI-TOF/MS (*Matrix Assisted Laser Desorption Ionization-Time of Flight/Mass Spectrometry*).* CrataBL* was added to saturated *α*-cyano-4-hydroxycinnamic solution, spotted onto a stainless steel MALDI target plate, and dried at room temperature before analysis. The calibrants used were insulin, ubiquitin, cytochrome C, and myoglobin. The mass spectrum was obtained on a Bruker Daltonics Microflex LT (*Billerica, USA*).

Protein concentration was evaluated as previously described [19] using bovine serum albumin as standard.

### 2.2. Animals and Experimental Design

Male, pathogen-free BALB/c mice (6-7 weeks old, 25-30 g) were obtained from the University of Sao Paulo. All mice received humane care in compliance with the “Guide for the Care and Use of Laboratory Animals” [20], and all experiments described were approved by the institutional review board of the University of Sao Paulo (Sao Paulo, Brazil), number 187/12.

**Figure 1:**
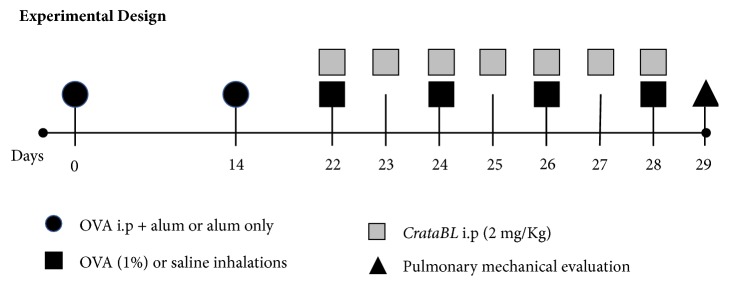
**Timeline of experimental design.** OVA and OVA+CR groups received OVA (50 *μ*g i.p.) + Alum (6 mg) on days 0 and 14, and on days 22, 24, 26, and 28 they were exposed to nebulized OVA aerosol. C and C+CR groups received only Alum i.p. on days 0 and 14, and on days 22, 24, 26, and 28 they received saline inhalations. C+CR and OVA+CR groups received treatment with* CrataBL* (2 mg/Kg i.p.) once daily (from day 22 to day 28; on inhalation days* CrataBL* was administered two hours after inhalation). On day 29, respiratory lung mechanics were assessed in all groups. Two independent experiments were conducted (n=8 per group per experiment).

Animals were divided into four groups: nonsensitized and nontreated mice (C group); ovalbumin- (OVA-) sensitized and nontreated mice (OVA group); nonsensitized and* CrataBL*-treated mice (C+CR group) and OVA-sensitized and* CrataBL*-treated mice (OVA+CR group) (Figure 1).

We conducted two independent experiments. In the first experiment, eight mice per group (n=8) were used to analyze hyperresponsiveness to methacholine, bronchoalveolar lavage, as well as for histopathological, morphometric, and immunohistochemistry analysis. In the second experiment, we also used n=8 mice in each group and performed hyperresponsiveness measurements in response to methacholine and ELISA for detection of cytokines IL-4, IL-5, and IFN-*γ* in lung homogenates. Data were pooled (n=16 per group) only for statistical analyses of hyperresponsiveness to methacholine.

### 2.3. Induction of Chronic Allergic Pulmonary Inflammation and* CrataBL* Treatment

The model of choice for allergic pulmonary inflammation induction was previously reported [21, 22] and is detailed in Figure 1. Animals were immunized intraperitonially on days 0 and 14 using 50 *μ*g of OVA (Grade IV, Sigma Aldrich, St. Louis, MO) with 6 mg of Al (OH)3 adjuvant (Pepsamar, Sandei-Synthelabo SA, Rio de Janeiro, Brazil) diluted in 0.2 mL saline. Eight days after the second immunization, the mice were placed in a Plexiglas box (30 x 15 x 20 cm) coupled to an ultrasonic nebulizer (US-1000, ICEL, São Paulo, Brazil), and an aerosol of OVA solution (1%) (Grade V, Sigma Chemical Co., St. Louis, MO) diluted in 0.9% sterile NaCl solution (saline) was generated for 30 minutes 4 times every other day. Control mice received the adjuvant intraperitoneally and were exposed to nebulized aerosol comprised of saline (0.9% NaCl).

Treated mice received intraperitoneal injections containing 2 mg/kg of* CrataBL* administered in 0.2 mL for each dose over seven consecutive days, two hours after inhalations exposures (days 22 to 28) (Figure 1). The administered dose was the same as that used in an experimental model of thrombosis [23], similar to the elastase inhibitor* BbCI (Bauhinia bauhinioides Cruzipain Inhibitor*) which is also a proteinase inhibitor used in asthma model [24, 25].

### 2.4. Evaluation of Pulmonary Mechanics and Bronchoalveolar Lavage Fluid

To determine whether* CrataBL* modulates hyperresponsiveness, 24 h after the last challenge (day 29, Figure 1), animals were anaesthetized with sodium pentobarbital (50 mg/kg), tracheostomized, and subsequently mechanically ventilated (Harvard 687, Harvard Apparatus, Holliston, MA) in an acrylic plethysmograph (120 cycles per minute, 10 mL/kg). Signs of tracheal pressure and lung volume were acquired through differential pressure transducers (Honeywell 163PC01D36, Freepot, IL) and converted to a digital analog board (DT01EZ, Data Translation, Marlboro, MA). Rrs and Ers of the respiratory system were calculated using the equation of motion: Ptr (t) = Rrs.V' (t) + Ers.V (t), where (t) is time, Ptr is tracheal pressure, Rrs is respiratory system resistance, Ers is respiratory system elastance, V' is airflow and V is the lung volume. Rrs and Ers values were obtained at baseline and after aerosol administration of methacholine (3, 30, and 300 mg/mL, for 1 min). We considered the maximal response of Rrs and Ers to in be in response to the 300 mg/mL methacholine challenge [21, 22].

After pulmonary responsiveness measurements, thorax of mice was opened, and animals were exsanguinated. BALF was performed by injecting a total of 1.5 mL saline (3 x 0.5 mL) through a tracheal cannula. The fluid collected was centrifuged at 790 x* g* for 10 min, at 5°C, and the cell pellet was resuspended in 0.3 mL of saline. From this solution, we placed 20 *μ*L on a* Neubauer* chamber with four quadrants to determine total cell counts. No dye was used, and the sum of this result was corrected for to 0.3 mL by multiplying by 10^4^. For differential cell counts, we took 100 *μ*L of the supernatant and placed it in the centrifuge (Cytospin) at 450 rpm for 6 minutes to obtain blades stained with Diff Quick. Three hundred cells were counted per slide, according to the standard morphologic criteria for differentiating polymorphonuclear cells, macrophages, and lymphocytes with assistance of an optical microscope with 1000x immersion objective [21, 22, 26].

### 2.5. Histopathological, Morphometric, and Immunohistochemistry Analysis

After pulmonary responsiveness measurements, mice were euthanized by vena cava dissection, the chest was opened, and the heart and lungs were removed* en bloc. *Lungs were fixed in 4% formalin in sufficient volume to achieve total immersion for 24 hours. After this period, the lungs were dehydrated in 70% ethanol followed by immersion in paraffin, and different regions of the lung were cut into slices to obtain 4-*μ*m thick sections that were then mounted onto slides. The slides were stained with hematoxylin-eosin for quantification of eosinophils, Resorcin-Fuchsin for analysis of elastic fibers, and with Picrosirius for analysis of collagen fibers (Direct Red 80, C.I. 35780, Aldrich, Milwaukee, WI, USA).

Additional slides were also prepared for immunohistochemical staining to assess protein expression using the following antibodies: interleukin 4 (IL-4, dilution 1:600, Santa Cruz Biotechnology, cod. SC-1260, Santa Cruz, CA, USA), interleukin 5 (IL-5, dilution 1:500, Santa Cruz Biotechnology, cod. SC-7887, Santa Cruz, CA, USA), interleukin 13 (IL-13, dilution 1:700, Santa Cruz Biotechnology, cod. SC-1776, Santa Cruz, CA, USA), interleukin 17 (IL-17, dilution 1:800, Santa Cruz biotechnology, SC-73309, Santa Cruz, CA, USA), interferon gamma (IFN-*γ*, dilution 1:200, Santa Cruz Biotechnology, cod. SC-8308, Santa Cruz, CA, USA), transforming growth factor beta (TGF-*β* dilution 1:100, Santa Cruz Biotechnology, cod. SC-1836, Santa Cruz, CA, USA, matrix metalloproteinase 9 (MMP-9, dilution 1:500, Santa Cruz Biotechnology, cod. SC-6840, Santa Cruz, CA, USA), 8-iso-PGF2*α* (dilution 1:10000, Oxford Biomedical Research, cod. IS20, Rochester Hills, MI, USA), tissue inhibitor of matrix metalloproteinase 1 (TIMP-1, dilution 1:200, Santa Cruz Biotechnology, cod. SC-5538, Santa Cruz, CA, USA), inducible nitric oxide synthase (iNOS, dilution 1:100, LabVision, NeoMarkers, cod. RB-9242, Fremont, CA, USA), and nuclear factor kappa B (NF*κ*B, dilution 1:100, Santa Cruz Biotechnology, cod. SC-109, Santa Cruz, CA, USA).

Slides containing tissue sections from the lungs were first deparaffinized and hydrated (Xylene 60°C Xylene 1, 2, and 3, first and second absolute ethanol, 96% ethanol, and 70% ethanol) followed by preparation with 3-aminopropyl triethoxysilane Silane (Sigma Aldrich, Missouri, USA). Slides were then washed with tap water and distilled water and subjected to the following procedures: antigen recovery; endogenous peroxidase blocking and nonspecific connections, incubation with primary antibody, incubation with secondary antibody, and peroxidase complex; visualization and counter-staining and mounting of slides [27, 28].

Conventional morphometric analysis was performed with an optical microscope using a point-counting technique [29] with a reticle of 50 lines and 100 points, totaling an area of 10^4^ *μ*m^2^. For evaluation of airways, the reticle was coupled adjacent to the wall of the airway in the bronchovascular axis from the base of the epithelium. Five airways were randomly selected for each animal, and approximately three fields per airway were evaluated. For evaluation of the alveolar septa, ten fields were randomly selected. This technique was used to quantify eosinophils density, volume fraction of isoprostane, and number of IFN-*γ*-, IL-4-, IL-5-, IL-13-, MMP-9-, TIMP-1-, TGF-beta-, iNOS-, and NF*κ*B-positive cells. Results are expressed as the number of cells per area (cells/10^4^ *μ*m^2^).

Homogeneity of slide samples for alveolar septa was assessed by measuring the fractional area of tissue constituents using the point-counting method with a 100-point grid with a known area (62,500 *μ*m^2^ at 400× magnification) attached to the ocular microscope. We measured the fractional area of the bronchial wall (BW), blood vessel wall (BVW), and alveolar wall (AW) as the number of points that fell in either the BW, BVW, or AW divided by the total number of points that fell within the strip tissue. Measurements were performed in 10 fields per slide at 400× magnification, and we calculated the mean values for each animal [27, 28].

### 2.6. Enzyme-Linked Immunosorbent Assay (ELISA)

ELISA was performed using the DuoSet kit for mouse (R&D System, Minneapolis, USA) according to the manufacturer's instructions for detection of cytokines IL-4, IL-5, and IFN-*γ* in lung homogenates. Microplates (Costar, Cambridge, MA, USA) for each cytokine were coated with specific monoclonal antibodies. After washing and distribution of the samples, antibodies were added that were specific for different cytokines conjugated to biotin. For analysis, a solution containing an enzyme conjugate of streptavidin-peroxidase and chromogenic substrate was added to each well. Optical density (OD) of the reaction was read at 450 nm in an M2 spectrophotometer (Spectramax L, Molecular Devices). Sample concentrations were calculated by linear regression from the ODs using standard curves obtained with recombinant cytokines, and the results are expressed as pg/mL.

### 2.7. Quantification of Collagen and Elastic Fibers Volume Fraction

To study the elastic fibers, sections were dewaxed and hydrated in 95% alcohol and subsequently stained with Weigert's Resorcin-Fuchsin with oxidation. There were two changes of 70 alcohol for 10 min to differentiate the fibers, which were then were dehydrated, diaphanous, and retrofitted. Fibers were counted in the airways, and the readings were quantified using an* Image Analyzer with *results expressed as the percentages of positive areas (volume fraction).

Picrosirius (Direct Red 80, C.I. 35780, Sigma Aldrich) staining was used to quantify collagen fibers in the airways. Sections were dewaxed and stained for 1 hour in Picrosirius at room temperature. Slices were then washed in running water for 5 min and after this step, and sections were stained with* Harris *hematoxylin for 6 min and washed in running water again for 10 min. The number of collagen fibers in the airways and in alveolar septa were counted and recorded using* Image Analyze, *and results are expressed as a percentage of the fiber-positive area (volume fraction) as described below.

Measurement of optical density was the method employed for analysis of elastic and collagen fibers. Images were acquired using a Leica DM4000B microscope (Leica Microsystems, Wetzlar, Germany) and a digital camera (Leica DFC420 Leica Microsystems) connected to a computer at a magnification of 400x. Ten airways and twenty fields of alveolar septa were captured from each animal. Threshold color tones were selected and represented the positive areas. In each slide, the analyzed field was selected, and the software performed quantitative analysis of the volume fraction of positive cells for each field (ImageProPlus-Media Cybernetics, Bethesda, MD). This calculation was performed for each analyzed field. Area considered positive, according to the predetermined threshold color tones, was divided by the total area [27, 28].

### 2.8. Passive Cutaneous Anaphylaxis

Passive cutaneous anaphylaxis (PCA) was performed for anti-OVA IgE and anti-OVA IgG1, as previously described [30, 31]. Seven days after the last inhalation of OVA, serum was collected from each group of animals and prepared at different dilutions. Then, naïve animals (Wistar rats and mice were used for anti-IgE and for anti-IgG1, respectively) had their backs shaved and were intradermally injected with different prepared dilutions. Mice and rats were IV challenged with 0.5 mg of OVA in 0.25% Evans Blue solution two and twenty-four hours later, respectively. PCA titer is expressed as the reciprocal of the highest dilution that produced an intradermic allergic reaction >5 mm in diameter in duplicate tests. Detection of threshold for this technique was established at a 1:5 [30, 31].

### 2.9. Data Analysis

The statistical analysis was performed using SigmaStat software (SPSS Inc., USA). Data are presented as the means ± standard errors (SEs). Statistical significance between groups was assessed using one-way analysis of variance (ANOVA) followed by the Holm-Sidak method for multiple comparisons. We also calculated the Pearson's correlation coefficient (*R*) to assess associations of respiratory system resistance (Rrs) and elastance (Ers) scores and inflammatory cell markers, remodeling, and oxidative stress markers. Differences were considered to be significant when P<0.05.

## 3. Results

### 3.1. Homogeneity of* CrataBL*

**Figure 2:**
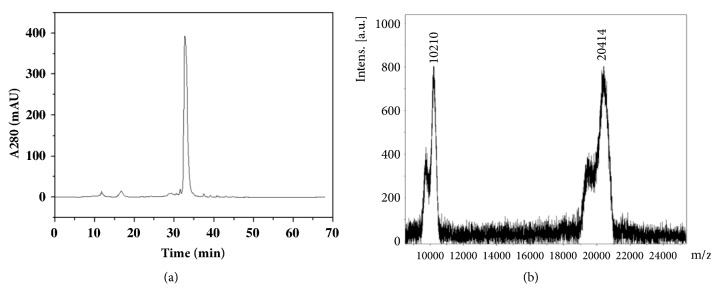
**Purified* CrataBL* detected by HPLC and mass spectrometry**. (a) Protein fraction was chromatographed on a C18 column eluted with a linear gradient (5–100%) of 90% acetonitrile in 0.1% TFA in Milli-Q water (solvent B) (t = 0.1 min, 5% B; t = 5 min, 5% B; t = 30 min, 40% B; t = 50 min, 50% B; t = 60 min, 100% B; t = 65–68 min, 0% B) at the flow rate of 0.7 mL/min. (b) MALDI-TOF mass spectrum of* CrataBL*.

The primary sequence and resolution of* CrataBL's* 3D structure were elucidated by Ferreira et al. [12].* CrataBL* homogeneity used in our study was confirmed by elution of one main fraction by reverse phase chromatography HPLC system (Figure 2(a)) and the mass spectrum profile (Figure 2(b)), which was similar to that previously reported [12] (20.414 (M+H)^+^ and 10210 due to molecular ion (M+2H)^+^). The 19.5-kDa isoform was also detected in the preparation used.

### 3.2. *CrataBL* Attenuates Hyperresponsiveness to Methacholine

**Figure 3:**
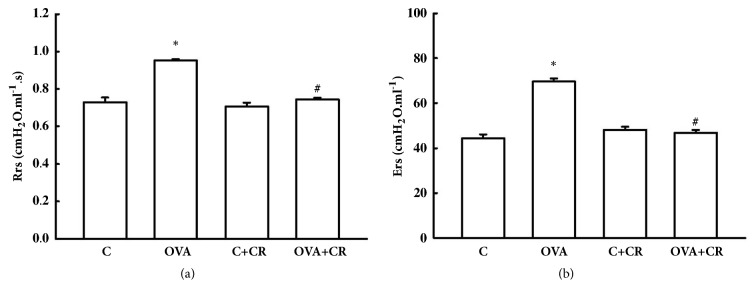
***CrataBL* attenuates hyperresponsiveness to methacholine. **Bar graph showing mean ± standard error values of the maximum response of respiratory system resistance (**a**) and elastance (**b**) after methacholine challenge (300 mg/mL) in the four experimental groups. Two independent experiments were conducted (n=8 per group per experiment) and for responsiveness measurements to methacholine, data were pooled, totaling n=16 per group. ^*∗*^P<0.05 compared to C and C+CR groups, #P<0.05 compared to OVA group.

The OVA group exhibited increased Rrs compared to controls (OVA group: 0.94±0.00* versus* C group: 0.74±0.02 or C+CR group: 0.71±0.02 cmH_2_O.mL^−1^.s, P<0.05). Rrs was significantly decreased in the OVA+CR group (0.75±0.01 cmH_2_O.mL^−1^.s) compared to the OVA group (P<0.05) (Figure 3(a)). Ers values after maximal dose of methacholine challenge in the OVA group were higher compared to controls (OVA group: 69.56±1.42* versus* C group: 44.41±1.74 or C+CR group: 48.20±1.20 cmH_2_O.mL^−1^, P<0.05). The OVA+CR group exhibited decreased Ers (46.89±1.28 cmH_2_O.mL^−1^) compared to the OVA group (P<0.05) (Figure 3(b)). These data suggest that* CrataBL* treatment ameliorates hyperresponsiveness.

### 3.3. *CrataBL* Attenuates Cells on BALF

**Figure 4:**
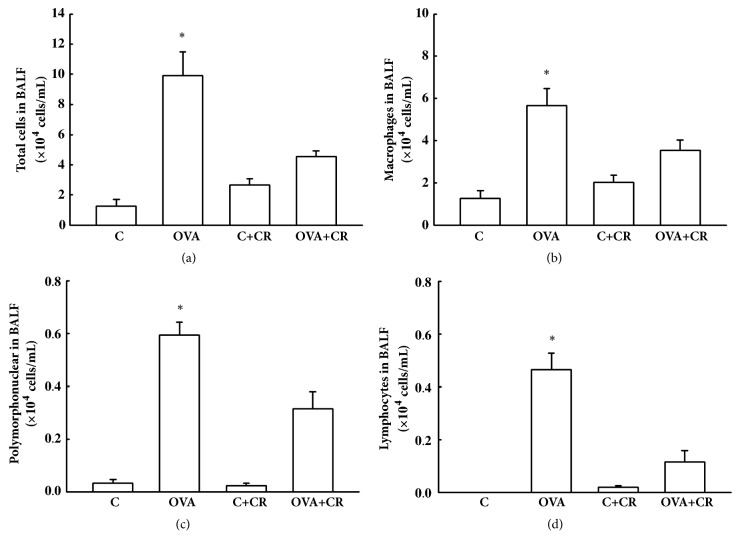
***CrataBL* attenuates inflammation in bronchoalveolar lavage fluid (BALF). **Bar graph showing mean ± standard error of total cells (a), macrophages (b), polymorphonuclear cells (c), and lymphocytes (d). Results are expressed in 10^4^ cells/mL. Eight animals per group were used for these analyses. ^*∗*^P<0.05 compared to OVA+CR, C and C+CR groups.

Results of BALF cells quantification are shown on Figure 4. The OVA group showed higher cell numbers on BALF compared to the controls (p<0.05). OVA+CR attenuated total cells number, including macrophages, lymphocytes, and polymorphonuclear cells, compared to the OVA group (p<0.05).

### 3.4. *CrataBL* Attenuates Inflammation, Oxidative Stress, and Extracellular Matrix Remodeling in Airway and Alveolar Walls

We observed increased IL-4, IL-5, and IFN-*γ* in lung homogenates in the OVA group compared to controls (P<0.05) as detected by ELISA. Treatment with* CrataBL* (OVA+CR group) reduced these values compared to the OVA group (P<0.05) (Table 1).

Tables 2 and 3 show inflammatory and remodeling markers as well as NF*κ*B positive cells in airways and alveolar walls of all groups.

The number of eosinophils in the airways (Table 2) of the OVA group were significantly higher than in controls (P<0.05). We also observed increased IL-4, IL-5, IL-13, IL-17, IFN-*γ*, NF*κ*B, MMP-9, TIMP-1, and TGF-*β* positive cells (P<0.05). Compared to the OVA group, OVA+CR resulted in a 68.3% significant reduction in the number of eosinophils, 56.3% in IL-4, 60.2% in IL-5, 79.2% in IL-13, 66.4% in IL-17, 63.3% in IFN-*γ*, 72.3% in NF-kB, 73.6% in MMP-9, 76.7% in TIMP-1, and 37.1% in TGF-*β* positive cells (P<0.05).

Furthermore, we observed increased eosinophils in alveolar walls (Table 3) of the OVA group compared to controls (P<0.05). The OVA group also exhibited increased IL-4, IL-5, IL-13, IL-17, IFN-*γ*, NF*κ*B, MMP-9, TIMP-1, and TGF-*β* positive cells compared to controls (P<0.05). Compared to the OVA group, the OVA+CR group had significantly reduced (56.8%) eosinophil numbers, 57.4% in IL-4, 68% in IL-5, 52.5% in IL-13, 48.9% in IL-17, 59.1% in IFN-*γ*, 61.3% in NF-kB, 62.3% in MMP-9, 48.5% in TIMP-1, and 48.7% in TGF-*β* positive cells (P<0.05).

For evaluation of elastic and collagen fiber content, we used measurement of optical density as described in the materials and methods. There were increased collagen and elastic fibers content in the airways of the OVA group compared to controls (P<0.05). The OVA+CR group had a 36.6% reduction in the volume of collagen fiber and of 52.4% reduction in elastic fibers compared to the OVA group (P<0.05) (Table 2). There was increased collagen and elastic fibers content in the alveolar wall in the OVA group compared to controls (P<0.05). The OVA+CR group had a 55.8% reduction in collagen fibers and of 31.5% reduction in the elastic fibers compared to the OVA group (P<0.05) (Table 3).

**Figure 5:**
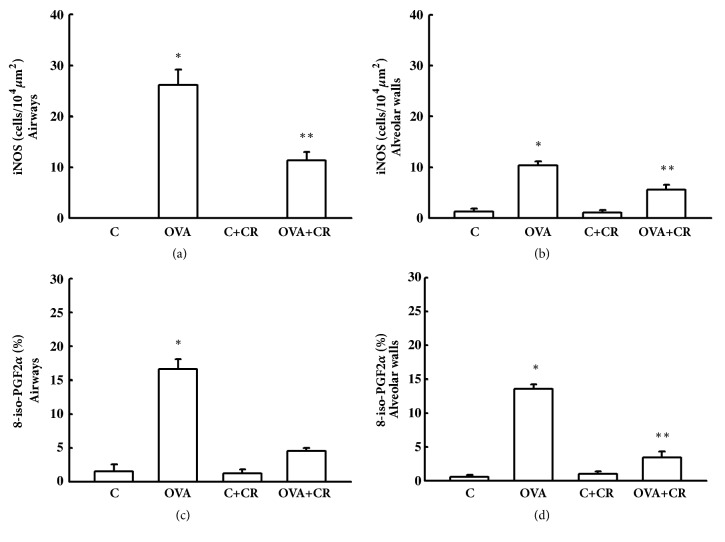
***CrataBL* attenuates oxidative stress markers in the airway and alveolar walls.** Bar graph showing mean ± standard error of iNOS in airway and alveolar walls (a and b, respectively) and the volume fraction of 8-iso-PGF2-*α* positive cells in airway and alveolar walls (c and d, respectively). Results are expressed in cells/10^4^*μ*m^2^. Eight animals per group were used for these analyses. ^*∗*^P<0.05 compared to OVA+CR, C and C+CR groups.

Figure 5 shows results for oxidative stress markers in the airways and alveolar walls. The OVA group exhibited increased iNOS and increased volume fraction of 8-iso-PGF2-*α* positive cells in both the airway and alveolar walls. Compared to the OVA group, the OVA+CR group had significantly decreased iNOS (Figures 5(a) and 5(b), for airway and alveolar walls, respectively) (P<0.05 for both) and significantly decreased volume fraction of 8-iso-PGF2-*α* positive cells (Figures 5(a) and 5(b), for airway and alveolar walls, respectively) (p<0.05).

These results suggest that* CrataBL* modulates inflammatory, remodeling and oxidative stress markers. The decreasing in NF-kB-positive cells may suggest one mechanism whereby attenuation of the inflammatory response occurs.

### 3.5. Qualitative Analysis of Photomicrographs

**Figure 6:**
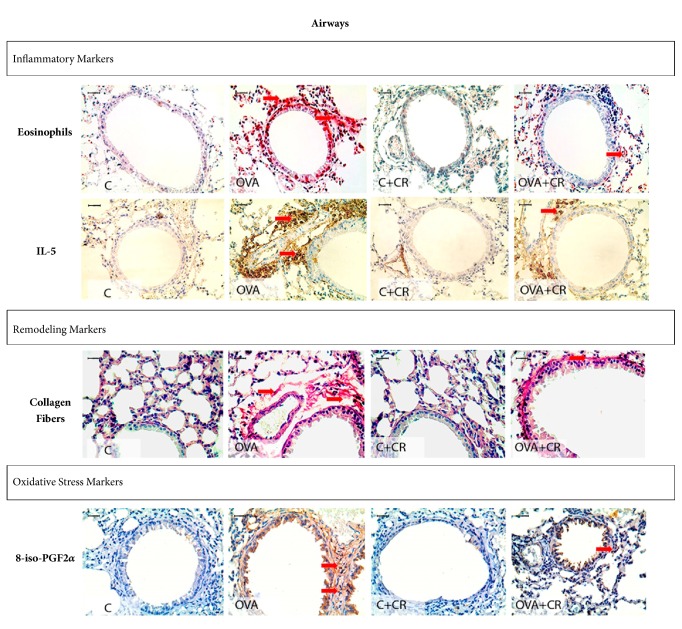
***CrataBL* attenuates inflammation, oxidative stress, and extracellular matrix remodeling in the airways. **Photomicrographs showing inflammatory markers: eosinophils and IL-5; remodeling markers: collagen fibers content and oxidative stress markers: 8-iso-PGF2*α* volume fraction in the airways. Scale bar represents 10 *μ*m. All experimental groups are represented (n=8 per group): C, OVA, C+CR, and OVA+CR groups.

**Figure 7:**
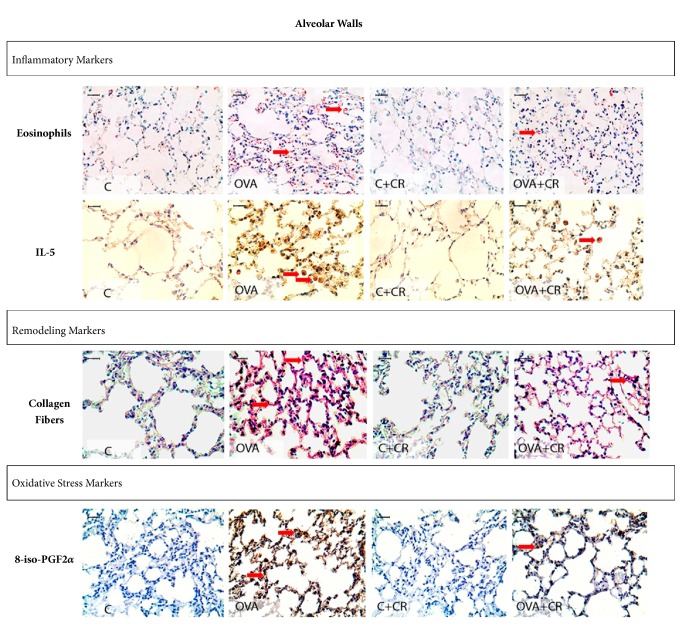
***CrataBL* attenuates inflammation, oxidative stress, and extracellular matrix remodeling in alveolar walls. **Photomicrographs showing inflammatory markers: eosinophils and IL-5; remodeling markers: collagen fibers content and oxidative stress markers: 8-iso-PGF2*α* volume fraction in the alveolar walls. Scale bar represents 10 *μ*m. All experimental groups are represented (n=8 per group): C, OVA, C+CR, and OVA+CR groups.

Microscopic analyses were performed at 400x magnification to highlight the differences among groups. Representative photomicrographs of inflammatory processes, extracellular matrix remodeling, and oxidative stress markers in the airways and alveolar walls are shown in Figures 6 and 7. The OVA group exhibited prominent increases in these makers in both the airways and alveolar walls compared to controls and the OVA+CR group.

### 3.6. Correlation Analysis

Correlation analysis of Rrs and Ers with the measurements of inflammatory, remodeling, and oxidative stress markers in the airways and alveolar walls are shown in Tables 4 and 5, respectively. Significant correlations between all parameters were observed.

### 3.7. OVA-Sensitized Animals Present IgG1 and IgE Antibodies

Results of the PCA test revealed increased OVA-specific IgE and IgG1 antibody titers in both OVA-sensitized groups (OVA group and OVA+CR group). The maximum titer for both groups was 1:1280 (for both, anti-OVA IgE and anti-OVA IgG1 antibodies).* CrataBL* treatment did not significantly alter OVA-specific IgE and IgG1 antibodies analyzed by PCA technique. As expected, there was no anti-OVA IgG1 or anti-OVA IgE detected in control groups (C and C+CR groups).

## 4. Discussion

Herein, we demonstrated that* CrataBL* treatment reduces hyperresponsiveness to methacholine and attenuates eosinophils, Th1/Th2/Th17 inflammatory cytokines, extracellular matrix remodeling, and NF*κ*B-positive cells. In addition,* CrataBL* treatment attenuated the number of iNOS-positive cells and the volume fraction of 8-iso-PGF2*α* content in the airways and alveolar walls in a model of allergic pulmonary inflammation.

*CrataBL* shows peculiar characteristics of being both a lectin and a proteinase inhibitor. In general, lectins are involved in numerous biological processes, including host-pathogen interactions and recruitment of leukocytes to inflammatory sites [32]. Lectins act by binding in the carbohydrate structures on the cell surface, whereas proteinase inhibitors act by limiting the catalytic activity of proteinases, which participate in many biological processes [33, 34]. These two properties have been studied in different biological processes, and the* CrataBL* inhibitor demonstrates important biological effects based primarily on its antiplatelet [14, 23], insecticide [16], and antitumor activities [12]. Furthermore, other studies suggest that another Kunitz-type plant proteinase inhibitor,* BbCl* (*Bauhinia bauhinioides cruzipain* inhibitor), exhibits anti-inflammatory and antioxidant pulmonary activities in experimental elastase-induced pulmonary inflammation in mice [25, 27]. There is also evidence of beneficial effects of proteinase inhibitors in asthma models [13, 35]. In this regard, Lin et al. reported the beneficial effects of serine proteinase inhibitors in the* Der p*-induced asthma model, resulting in decreased airway hyperresponsiveness and remodeling, eosinophil and neutrophil infiltration, the Der p-induced IL-4 levels in serum, and Th2 and Th17 cytokines profiles in BALF [13]. However, there is still controversy concerning proteinases and their specific functions that contribute to the development and progression of asthma. It is also important to remember that many allergens are proteinases, which may help explain the influence of proteinase inhibitors on asthma [35, 36].

The* CrataBL* inhibitor was administered to the OVA+CR group after sensitization [37]. PCA analysis showed that only OVA-sensitized animals (OVA and OVA+CR groups) exhibited increased OVA-specific IgE and IgG1 antibody titers (1:1280) and no difference was observed between them, suggesting that* CrataBL* treatment (OVA+CR group) did not interfere in pathogenic, IgE-generating memory B cell responses.

The methacholine challenge is commonly employed to quantify hyperresponsiveness [38]. Maximum increases in Rrs and Ers after methacholine challenge in the OVA group observed in our study are similar to other experimental asthma models [21, 31, 39].* CrataBL* treatment of OVA-sensitized mice (OVA+CR group) reduced Rrs and Ers to methacholine challenge compared to the OVA group, suggesting a bronchodilator effect of the* CrataBL* proteinase inhibitor. In addition, we observed that treatment with* CrataBL* in OVA-sensitized mice (OVA+CR group) attenuated inflammation, remodeling, and oxidative stress markers in both airways and alveolar walls (Tables 2 and 3 and Figure 5), contributing to understanding the potential mechanism of action of the* CrataBL* proteinase inhibitor. Our results are similar to those that used other proteinase inhibitors to target pulmonary inflammation, remodeling, and hyperresponsiveness in animal models [13, 25, 27, 28].

In the current study, inflammatory response assessed by BALF analysis showed that the OVA+CR group had significantly reduced total cell numbers, including decreased macrophages, polymorphonuclear and lymphocytes, compared to the OVA group, suggesting an important role for* CrataBL* as an anti-inflammatory protein. Neuhof* et al*. (2003) found that pulmonary edema in isolated rabbit lungs caused by neutrophil elastase was significantly decreased in response to* BbCI* [40]. Moreover, Oliveira et al. [24] used inflammation models in pretreated rats with* BbCI* and observed a reduction in leukocyte rolling, adhesion, and migration in inflamed tissues. This observation may be due to* BbCI* potent inhibitory effect on three proteinases that play important roles in the inflammatory processes: human leukocyte elastase (HLE), cathepsin G, and cathepsin L [24]. In contrast, there is currently no evidence of direct inhibition by* CrataBL* on the activity of HLE or cathepsin G. However,* CrataBL* may significantly interfere in these processes after binding to heparin and glycosaminoglycans on the cell surface [41]. Heparin potentiates proteinase activity, primarily in cathepsins [42] and plasma kallikrein [43]; thus,* CrataBL* binding to heparin may interfere with proteolytic activity and generation of proinflammatory peptides, such as kinins (e.g., bradykinin). This indirect interference in proteolytic activity also correlates to our findings of decreased metalloproteinases levels (Tables 2 and 3).

In addition, eosinophils were increased in the airways and alveolar septa in the OVA group, and* CrataBL* treatment significantly mitigated this response in the OVA+CR group. Furthermore, we observed that* CrataBL* treatment attenuated numbers of Th1 (IFN-*γ* positive cells), Th2 (IL-4, IL-5, and IL-13) and Th17 (IL-17) positive cells in the airways and alveolar walls. These results suggest that* CrataBL* modulates signaling events involved in the recruitment of eosinophils and lymphocytes during an allergic inflammatory response. It is noteworthy to reiterate that the most dramatic effect was noted in cellular expression of IL-5 in the alveolar walls (68% reduction), which is related to eosinophil chemotaxis [44].

In our study, we observed a significant reduction in collagen and elastic fiber volume fraction in OVA-sensitized and* CrataBL-*treated mice (OVA+CR group) compared to the OVA group. This observation is likely explained by the binding of* CrataBL* to heparin, which interferes with its ability to enhance proteinases activities. Therefore,* CrataBL* may be effective in blocking activation of proteinases and in the breakdown of elastic fibers located within airways and alveolar walls. A similar response was observed in studies using a direct inhibitor* (BbCl)* [25].

Metalloproteinase expression, especially MMP-9, seems to play an important role in the asthma remodeling process [45]. In our study, we found significant increases in MMP-9 in airways and alveolar walls in the OVA group and significant decreases in the OVA+CR group. In addition, cathepsins have high affinity for heparin and can activate prourokinase that is responsible for activating plasminogen to form plasmin, which in turn activates MMP-2 and MMP-9 metalloproteinases. Plasmin further increases metalloproteinase and cathepsin activities. We speculate that the effect of* CrataBL *in the coagulation intrinsic pathway [14] may interfere with heparin's role in amplifying the network of proteinase activation in pathophysiological processes. Involvement of coagulation factors and the fibrinolytic system and platelets in the pathophysiology of asthma is being studied by others [46].

We also observed an increase in TIMP-1 in the airways and alveolar walls in the OVA group. There is evidence suggesting that this may occur in an attempt to repair damage [47]. It is important to note that, besides TIMP-1 being a specific tissue inhibitor of MMP-9, it has also been recognized to have biological effects independent of MMPs, such as antiapoptotic activity [48]. In our study, we observed a reduction in the number of MMP-9-, TIMP-1-, and TGF-*β*-positive cells and reduced volume fractions of collagen and elastic fibers in the OVA+CR group, which were most likely due to inhibition of that pathway. Furthermore, it has been shown that the reduction of eosinophils from treatment with anti-IL-5 is associated with a significant decrease in expression of proteins in the extracellular matrix, suggesting that TGF-*β* can regulate remodeling [49].

Production of reactive nitrogen and oxygen species denotes activity and effects of disease progression. Nitric oxide (NO) and isoprostanes are considered biomarkers of oxidative stress, and high levels of these substances may amplify deleterious and harmful effects on the lungs [50]. Interestingly, the reduction in isoprostane by* CrataBL*, with consequent decreases in peroxynitrite and isoprostane PGF-2*α* formation, suggests that this natural protein protects the cell membrane from lipid peroxidation.

In the present study, we observed that attenuated mechanical responses were associated with significant decreases in iNOS-positive cells. The reduction in the oxidative stress by treatment with* CrataBL* could also have contribute to attenuating remodeling. In this regard, Prado et al. [51] showed that iNOS inhibition reduced MMP-9, TIMP-1, and TGF-*β*-positive cells. These mediators participate in the production of collagen and elastic fibers, thus contributing to remodeling. Regarding the response in the airway and alveolar walls from animals with chronic allergic pulmonary inflammation, iNOS inhibitors (1400W or L-NAME) attenuated the response to antigen challenge. This functional impairment was associated with control of inflammation, remodeling, and oxidative stress [52].

Thus, in the inflammation process, activation of proteinases leads to increased production of bradykinin and subsequent activation of proinflammatory cytokines, which increase numbers of cNOS- and iNOS-positive cells. As previously mentioned,* CrataBL* neutralizes the effects of heparin, which may lead to a decrease in the potentiation of plasma kallikrein activity by this glycosaminoglycan, reducing kinin generation by kallikrein or other serine proteinases [23, 41]. These results are important in furthering our understanding of mechanisms for various diseases in which proteolytic activity has an important function [53].

NF*κ*B is an important modulator of inflammation in pulmonary diseases [54], and in the release of proinflammatory cytokines and chemokines. We found that NF*κ*B was reduced in the OVA+CR group compared to untreated animals, and some studies have shown an association between NF*κ*B activation and inflammation, oxidative stress, and remodeling in chronic pulmonary inflammation [55].

There was a strong correlation between changes in Rrs and Ers in the presence of inflammation, oxidative stress, and extracellular matrix remodeling. This positive correlation indicated that the activation of these signaling pathways was able to promote functional changes in both airways and alveolar walls.

There are some limitations of this study. First, we did not evaluate the dose-response curve of* CrataBL*. Second, although we did not lose any* CrataBL*-treated mice, there was no evaluation of the toxicity profile of* CrataBL*. However, experimental studies on the effect of high protein concentrations (2-6 mg/kg) on animal mortality were performed, and no effect on animal survival or behavior was observed [23]. Third, the potential mechanisms of action of* CrataBL* were not directly tested in our study. It is important to note that even though* CrataBL* is a natural compound of plant origin with proteinase inhibitor activity, it is likely not safe to extrapolate these findings to human beings. Furthermore, additional preclinical studies evaluating these issues and mechanism of action are necessary before taking this treatment strategy to clinical studies in human beings.

## 5. Conclusion

In this experimental model of allergic pulmonary inflammation, treatment with* CrataBL* decreased airway constriction, resulting in a significant attenuation of inflammatory responses, extracellular remodeling markers, and decreased oxidative stress response in the airways and alveolar walls. These results suggest that* CrataBL* plays an important role in controlling asthma. Further development of this treatment strategy is warranted before it can be developed as an additional therapeutic tool for controlling asthma.

## Figures and Tables

**Table 1:** Mean ± standard error values in ELISA analysis for IL-4, IL-5, and IFN-*γ* in the four experimental groups. Eight animals per group were used in these analyses. ^*∗*^P<0.05, compared to OVA+CR, C, and C+CR groups; **†** P<0.05 compared to C and C+CR groups.

**ELISA**	**C**	**OVA**	**C+CR**	**OVA+CR**
**IL-4 (pg/mL)**	86.01±8.71	308.00±35.80^*∗*^	112.50±18.43	156.82±11.73
**IL-5 (pg/mL)**	45.72±18.42	160.04±20.60^*∗*^	38.90±20.16	66.40±9.99
**IFN-** **γ** ** (pg/mL)**	94.85±13.04	456.96±32.87^*∗*^	94.46±12.52	324.47±11.19 **†**

**Table 2:** Absolute values of morphometric and optical density measurement analysis for inflammatory, remodeling, and oxidative stress markers in the airways. Eight animals per group were used in these analyses. ^*∗*^P<0.05 compared to OVA+CR, C, and C+CR groups; **† **P<0.05 compared to C and C+CR groups.

**Inflammatory Markers**	**C**	**OVA**	**C+CR**	**OVA+CR**
**Eosinophils (cells/10** ^**4**^ ***μ*** **m** ^**2**^ **)**	0.23±0.14	19.72±1.34^*∗*^	0.14±0.14	6.24±1.31 **†**
**IL-4 (cells/10** ^**4**^ ***μ*** **m** ^**2**^ **)**	2.19±0.10	21.74±2.08^*∗*^	1.19±0.78	9.49±0.93 **†**
**IL-5 (cells/10** ^**4**^ ***μ*** **m** ^**2**^ **)**	0.62±0.40	19.82±1.47^*∗*^	0.74±0.74	7.88±0.67 **†**
**IL-13 (cells/10** ^**4**^ ***μ*** **m** ^**2**^ **)**	2.19±0.47	23.18±1.92^*∗*^	1.47±0.74	4.82±0.61
**IL-17 (cells/10** ^**4**^ ***μ*** **m** ^**2**^ **)**	1.04±0.30	7.83±0.70^*∗*^	1.33±0.23	2.63±0.33**†**
**IFN-** **γ** ** (cells/10** ^**4**^ ***μ*** **m** ^**2**^ **)**	1.64±0.68	16.39±1.22^*∗*^	1.30±0.61	6.02±1.21 **†**

**Remodeling Markers**	**C**	**OVA**	**C+CR**	**OVA+CR**

**Collagen Fibers (%)**	7.73±1.27	18.90±1.31^*∗*^	5.26±0.69	11.98±0.75 **†**
**Elastic Fibers (%)**	6.50±0.55	16.43±1.04^*∗*^	5.73±0.68	7.81±0.76
**MMP-9 (cells/10** ^**4**^ ***μ*** **m** ^**2**^ **)**	1.30±0.89	18.30±2.59^*∗*^	0.00±0.00	4.82±0.61
**TIMP-1 (cells/10** ^**4**^ ***μ*** **m** ^**2**^ **)**	0.41±0.41	17.21±1.77^*∗*^	0.69±0.45	4.00±1.14 **†**
**TGF-** **β** ** (cells/10** ^**4**^ ***μ*** **m** ^**2**^ **)**	0.54±0.54	24.34±1.62^*∗*^	0.43±0.43	15.29±1.06 **†**

**Mechanism**	**C**	**OVA**	**C+CR**	**OVA+CR**

**NF-** **κ** **B (cells/10** ^**4**^ ***μ*** **m** ^**2**^ **)**	1.13±0.71	16.62±2.42^*∗*^	1.19±0.54	4.59±0.64

**Table 3:** Absolute values of morphometric and optical density measurement analysis for inflammatory, remodeling, and oxidative stress markers in the alveolar walls. Eight animals per group were used for these analyses. ^*∗*^P<0.05 compared to OVA+CR, C and C+CR groups; **† **P<0.05 compared to C and C+CR groups.

**Inflammatory Markers**	**C**	**OVA**	**C+CR**	**OVA+CR**
**Eosinophils (cells/10** ^**4**^ ***μ*** **m** ^**2**^ **)**	0.34±0.21	5.67±0.60^*∗*^	0.30±0.18	2.45±0.70 **†**
**IL-4 (cells/10** ^**4**^ ***μ*** **m** ^**2**^ **)**	0.61±0.20	12.27±0.82^*∗*^	0.54±0.39	5.22±0.71 **†**
**IL-5 (cells/10** ^**4**^ ***μ*** **m** ^**2**^ **)**	0.95±0.38	13.76±0.93^*∗*^	1.03±0.49	4.40±0.57 **†**
**IL-13 (cells/10** ^**4**^ ***μ*** **m** ^**2**^ **)**	0.90±0.44	7.71±1.21^*∗*^	0.48±0.32	3.66±1.49
**IL-17 (cells/10** ^**4**^ ***μ*** **m** ^**2**^ **)**	1.25±0.31	12.20±0.91^*∗*^	1.10±0.06	6.23±0.55**†**
**IFN-** **γ** ** (cells/10** ^**4**^ ***μ*** **m** ^**2**^ **)**	0.90±0.44	14.50±1.34^*∗*^	0.93±0.43	5.92±1.30 **†**

**Remodeling Markers**	**C**	**OVA**	**C+CR**	**OVA+CR**

**Collagen Fibers (%)**	2.97±0.29	12.89±0.90^*∗*^	2.88±0.40	5.69±0.70 **†**
**Elastic Fibers (%)**	5.52±0.37	12.15±0.47^*∗*^	5.78±0.25	8.32±0.43 **†**
**MMP-9 (cells/10** ^**4**^ ***μ*** **m** ^**2**^ **)**	0.68±0.46	9.32±1.18^*∗*^	1.03±0.48	3.51±0.86
**TIMP-1 (cells/10** ^**4**^ ***μ*** **m** ^**2**^ **)**	0.68±0.46	9.32±1.18^*∗*^	1.03±0.48	4.80±1.30 **†**
**TGF-** **β** ** (cells/10** ^**4**^ ***μ*** **m** ^**2**^ **)**	0.66±0.41	13.51±0.93^*∗*^	0,64±0.43	6.92±0.90 **†**

**Mechanism**	**C**	**OVA**	**C+CR**	**OVA+CR**

**NF-** **κ** **B (cells/10** ^**4**^ ***μ*** **m** ^**2**^ **)**	0.58±0.40	10.02±0.87^*∗*^	0.42±0.26	3.88±0.75 **†**

**Table 4:** Pearson Correlation coefficients of Rrs and Ers with the results of inflammatory, remodeling, and oxidative stress markers in BALF, ELISA, and airways.

	**Rrs**	**Ers**
	***Valor de R***	***Valor de R***
	***Valor de p***	***Valor de p***
**Total cells (BALF)**	0.863	0.918
	0.000302	0.0000256
**Macrophages (BALF)**	0.827	0.894
	0.000905	0.0000878
**Polymorphonuclear (BALF)**	0.920	0.961
	0.0000228	0.000000625
**Lymphocytes (BALF)**	0.845	0.895
	0.000536	0.0000823
**Eosinophils**	0.891	0.904
	0.000103	0.0000547
**IL-4 (ELISA)**	0.873	0.911
	0.000208	0.0000387
**IL-4 **	0.918	0.929
	0.0000259	0.0000124
**IL-5 (ELISA)**	0.799	0.828
	0.00184	0.000887
**IL-5**	0.877	0.913
	0.000179	0.0000346
**IL-13**	0.893	0.947
	0.0000905	0.00000290
**IFN-** **γ** ** (ELISA)**	0.806	0.759
	0.00156	0.00421
**IFN-** **γ**	0.937	0.954
	0.00000725	0.00000147
**Collagen Fibers **	0.868	0.897
	0.000248	0.0000776
**Elastic Fibers **	0.946	0.949
	0.00000337	0.00000256
**MMP-9**	0.908	0.934
	0.0000435	0.00000860
**TIMP-1**	0.903	0.948
	0.0000562	0.00000273
**TGF-** **β**	0.869	0.857
	0.000243	0.000374
**iNOS**	0.889	0.902
	0.000112	0.0000594
**8-iso-PGF2** **α**	0.931	0.950
	0.0000112	0.00000233
**NF-** **κ** **B **	0.913	0.936
	0.0000345	0.00000737

**Table 5:** Pearson correlation coefficients of Rrs and Ers with the results of inflammatory, remodeling, and oxidative stress markers in the alveolar walls.

	**Rrs**	**Ers**
	***Valor de R***	***Valor de R***
	***Valor de p***	***Valor de p***
**Eosinophils**	0.806	0.799
	0.00156	0.00184
**IL-4 **	0.904	0.928
	0.0000546	0.0000139
**IL-5**	0.877	0.929
	0.000179	0.0000127
**IL-13**	0.869	0.858
	0.000240	0.000356
**IFN-** **γ**	0.893	0.892
	0.0000904	0.0000949
**Collagen Fibers **	0.910	0.958
	0.0000397	0.000000980
**Elastic Fibers **	0.887	0.927
	0.000118	0.0000140
**MMP-9**	0.864	0.933
	0.000286	0.00000940
**TIMP-1**	0.874	0.911
	0.000202	0.0000376
**TGF-** **β**	0.915	0.915
	0.0000301	0.0000311
**iNOS**	0.827	0.849
	0.000905	0.000480
**8-iso-PGF2** **α**	0.890	0.935
	0.000105	0.00000821
**NF-** **κ** **B **	0.876	0.911
	0.000189	0.0000371

## Data Availability

The original data that support our results are available upon request to the corresponding author.
